# How to catch the N – An inter‐species exchange with the right chemistry

**DOI:** 10.15252/msb.20209514

**Published:** 2020-06-03

**Authors:** Tonni Grube Andersen

**Affiliations:** ^1^ Max Planck Institute for Plant Breeding Research Cologne Germany

**Keywords:** Metabolism, Microbiology, Virology & Host Pathogen Interaction, Plant Biology

## Abstract

While classical breeding traits have focussed on above‐ground tissues, it is becoming clear that underground aspects of plant life are a hidden treasure of tools applicable for resilient crop production. Plants of the legume family develop specialized organs, called nodules, which serve as hosts for *Rhizobium* bacteroids. A highly specialized symbiotic relationship exists deep inside the nodules. In exchange for carbohydrates, host‐specific rhizobia bacteroids can assimilate nitrogen from the air and fix it into a form that can be used by plants in a process known as biological nitrogen fixation. While we understand certain aspects of how this inter‐species relationship is established, the exact biochemistry of this exchange remains dogmatic. In their recent work, Christen and colleagues (Flores‐Tinoco *et al*, 2020) challenge the current model of nitrogen exchange and argue that that an expanded model is needed to fit experimental findings related to nitrogen fixation. The authors perform an elegant set of experiments and highlight that rather than a single‐way flow of nitrogen, the N‐fixing process is in fact an elaborate metabolic exchange between the nodule‐dwelling bacteroids and the host plant. Importantly, this work provides an updated theoretical framework with the “catchy” name CATCH‐N which delivers up to 25% higher yields of nitrogen than classical models and is suitable for rational bioengineering and optimization of nitrogen fixation in microorganisms.

## Graphical abstract

The biochemistry of nitrogen fixation in rhizobia‐plant symbiosis has remained dogmatic. In their recent work, Christen and colleagues (Flores‐Tinoco *et al*, 2020) show that rather than a single‐way N flow, N‐fixation involves an elaborate metabolic exchange between the bacteroids and the plant.

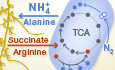

To date, the production of nitrogen‐containing artificial fertilizers still occurs by binding atmospheric N_2_ through the energy‐demanding Harber–Bosch reaction discovered more than a century ago (Appl, 1982). The prospect of implementing nitrogen fixation into non‐legume crops has fascinated researchers and breeders alike for centuries as this would decrease the demand for artificial fertilization. Therefore, in the emerging age of biotechnological solutions, one of the most intriguing features for agricultural improvement is to modify the crop of interest to either facilitate biological nitrogen fixation directly, or establish microbial relationships with natural or modified nitrogen‐fixing bacteria that would result in a net nitrogen gain for the plant. To catalyse atmospheric nitrogen fixation, rhizobia use a specific enzyme termed nitrogenase, which is able to catalyse formation of ammonium (Hoffman *et al*, 2014). This occurs under anaerobic conditions established inside the nodules (Oelze, 2000). Our current model of nutrient exchange in rhizobia–legume symbiosis postulates that, in exchange for nitrogen, the plant provides C4‐dicarboxylic acids such as succinate. These are then metabolized through the tri‐carboxylic acid (TCA) cycle to generate ATP and reduction equivalents needed for the nitrogenase reaction (Hoffman *et al*, 2014). In their work, Flores‐Tinoco *et al* (2002) highlight that several experimental discrepancies argue against such a model. For example, while the anaerobic environment encountered by rhizobia inside root nodules promotes nitrogenase activity, it also inhibits the catabolism of succinate through the TCA cycle. This goes hand in hand with the observation that increased levels of reductive co‐factors, such as NADH and NADPH, inhibit key enzymes of the TCA cycle (Dunn, 1998; Prell & Poole, 2006). In support of this, the authors also point out that nitrogen‐fixing bacteroids indeed secrete the amino acids alanine and aspartate (Kretovich *et al* 1986; Waters *et al* 1998; Allaway *et al* 2000) as expected from a partially operating TCA cycle.

Through a set of elegant experiments that include feeding of TCA intermediates and arginine to bacteroids and consecutive measurement of nitrogenase activity and ATP generation, the authors reveal that these factors are indeed boosted by the presence of an N‐source in the form of arginine. By feeding isotope‐labelled arginine to bacteroids, they analyse downstream catabolism and subsequently perform a transposon‐based mutant screening to identify bacterial catabolic mutants, which are then corroborated with low nitrogen plant phenotypes. Remarkably*,* this network is governed by highly redundant functions comprised of at least 10 transporter systems and 23 enzymatic functions (Flores‐Tinoco *et al*). Strikingly, the authors are able to identify these redundant bacterial enzymes participating in arginine transamination and—of particular note—are capable of reconstituting this complex network in vitro. Combining their experimental data, they create an updated framework for nitrogen assimilation termed C4‐dicarboxylate arginine transamination co‐catabolism under acidic (H^+^) conditions to fix nitrogen or “CATCH‐N” for short (Fig 1).

**Figure 1:**
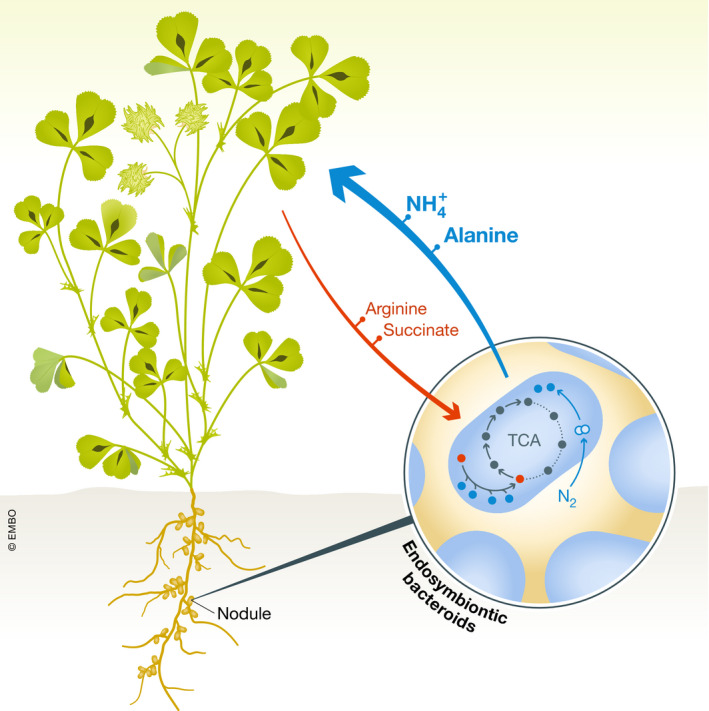
**The figure illustrates the** **CATCH** **‐N cycle, a metabolic pathway that co‐catabolizes arginine and succinate provided by the plant to drive symbiotic nitrogen fixation in endosymbiotic rhizobia.**

This interesting work uncovers an important biochemical principle of metabolic inter‐species interaction leading to the nitrogen‐fixing symbiosis between plants and bacteria. It emphasizes that this interplay is based on the co‐catabolism of plant‐provided arginine and succinate as part of a specific metabolic network. This sustains symbiotic nitrogen fixation as an interwoven but balanced two‐way exchange of C4‐dicarboxylate, protons and amino acids. The outcome yields a net gain in nitrogen for the plant and supports bacterial metabolism. This is an important stepping stone for the rational biotechnological engineering of artificial nitrogen‐fixing microbes and improved crop plants that are needed to ensure food and climate security.
